# Problematizing assumptions about interdisciplinary research: implications for health professions education research

**DOI:** 10.1007/s10459-019-09911-7

**Published:** 2019-08-20

**Authors:** Mathieu Albert, Farah Friesen, Paula Rowland, Suzanne Laberge

**Affiliations:** 1 Wilson Centre and Departement of Psychiatry, University of Toronto, Toronto, ON Canada; 2 Centre for Faculty Development, Faculty of Medicine, University of Toronto at St. Michaels Hospital, Toronto, ON Canada; 3 Wilson Centre, University of Toronto, Toronto, ON Canada; 4 Département de kinésiologie, Université de Montréal, Montréal, QC Canada

**Keywords:** Interdisciplinarity, Disciplines, Health professions education research

## Abstract

This article critically examines three assumptions underlying recent efforts to advance interdisciplinary research—defined in this article as communication and collaboration between researchers across academic disciplines (e.g. Sociology, Psychology, Biology)—and examines these assumptions’ implications for health professions education research (HPER). These assumptions are: (1) disciplines are silos that inhibit the free flowing of knowledge across fields and stifle innovative thinking; (2) interdisciplinary research generates a better understanding of the world as it brings together researchers from various fields of expertise capable of tackling complex problems; and (3) interdisciplinary research reduces fragmentation across groups of researchers by eliminating boundaries. These assumptions are among the new beliefs shaping the contemporary academic arena; they orient academics’ and university administrators’ decisions toward expanding interdisciplinary research and training, but without solid empirical evidence. This article argues that the field of HPER has largely adopted the premises of interdisciplinary research but has not yet debated the potential effects of organizing around these premises. The authors hope to inspire members of the HPER community to critically examine the ubiquitous discourse promoting interdisciplinarity, and engage in reflection about the future of the field informed by evidence rather than by unsubstantiated assumptions. For example: Should research centres and graduate programs in HPER encourage the development of interdisciplinary or disciplinary-trained researchers? Should training predominantly focus on methods and methodologies or draw more on disciplinary-based knowledge? What is the best route toward increasing the field’s profile within academia and attracting the best students and researchers to engage in HPER? These are questions that merit attention at the current juncture as the future of the HPER field relies on decisions made in the present time.

## Introduction

In this article, we critically examine three assumptions underlying recent efforts to advance interdisciplinary research—defined in this article as communication and collaboration between researchers across academic disciplines (e.g. Sociology, Psychology, Biology)—and examine these assumptions’ implications for health professions education research (HPER). These assumptions are: (1) disciplines are silos that inhibit the free flowing of knowledge across fields and stifle innovative thinking (Giacomini 2004; Jacob 2015); (2) interdisciplinary research generates a better understanding of the world as it brings together researchers from various fields of expertise capable of tackling complex problems (National Academy of Sciences 2005; Townsend et al. 2015); and (3) interdisciplinary research reduces fragmentation across groups of researchers by eliminating boundaries (Armstrong 2006; Giacomini 2004). These assumptions are among the new beliefs shaping the contemporary academic arena; they orient academics’ and university administrators’ decisions toward expanding interdisciplinary research and training, but without solid empirical evidence (Brint 2005; Frickel et al. 2017; Sá 2008). In Table 1, we present quotes from the academic literature exemplifying these three assumptions.

**Table 1:** The three pro-interdisciplinary assumptions used in this paper and representative quotes from the literature that support those assumptions

Assumptions	Representative quotes from academic literature
Assumption 1: Disciplines are silos that inhibit the free flowing of knowledge across fields and stifle innovative thinking	“Disciplinary models tend to prejudice researchers against seeing anything unexpected. (…) Disciplines create islands of knowledge (…); isolated specialists become unable to recognise relevant advances in parallel fields” (Giacomini 2004, p. 179) “The silo syndrome that permeates so many higher education institutions worldwide at the very least discourages interdisciplinary practices and at the most eliminates them from happening all together. A paradigm shift is needed to help provide an interdisciplinary enabling environment to encourage and facilitate interdisciplinary research” (Jacob 2015, p. 2)
Assumption 2: Interdisciplinary research generates a better understanding of the world as it brings together researchers from various fields of expertise capable of tackling complex problems	“In interdisciplinary research there is an assumption of interdependence, in that the theories, perspectives, tools and findings of one discipline cannot solve or illuminate the problem it is trying to solve so there is a sharing of purpose and methods (…)” (Townsend et al. 2015, p. 660) “Interdisciplinary research and education are inspired by the drive to solve complex questions and problems, (…) and lead researchers in different disciplines to meet at the interface and frontiers of those disciplines (…)” (National Academy of Sciences 2005, p. 16)
Assumption 3: Interdisciplinary research reduces fragmentation across groups of researchers by eliminating boundaries	“An interdisciplinarity dream is to unite scholars not only by getting them off their island to mix with each other but also by removing their blinders. Fundamental disciplinary goals, methods and values must be set aside” (Giacomini 2004, p. 179) … “a lucid summary of the factors that restrain interdisciplinary health research [begins] with the traditional structures, organizational matrix and culture of university faculties and departments. When this mix is garnished with the time-honoured territorial boundaries of professions, culturally coloured by their unique identities and lexicon, it is unsurprising that a unidimensional framework emerges” (Armstrong 2006, p. 761)

Surprisingly, while lively debates about academic disciplines and interdisciplinarity have been ongoing for decades outside HPER, starting as far back as the early 1970s (Organization for Economic Cooperation and Development 1972), the HPER community has been silent on this topic. A search in Web of Science for articles on inter-, multi-, and trans-disciplinarity in HPER revealed that the terms interdisciplinary or multidisciplinary are most often used to refer to inter-/multi-*professional* interactions (e.g. social workers and physicians) or inter-/multi-medical *specialization* interactions (e.g. orthopedic surgeons and neurosurgeons). Sometimes, a term like multidisciplinary is used without definition at all. This suggests there is an implicit understanding of what inter/multi/transdisciplinarity means within HPER. Yet this definition may not align with wider use of these terms across academia, where inter/multi/transdisciplinarity refers to interaction between academic disciplines, not between professions.

A recently published essay by van Enk and Regehr (2018) on the relationship between disciplinary-trained academics and health professionals in HPER stands out as the first article discussing knowledge production with an interdisciplinary lens. In their paper, van Enk and Regehr argue that HPER should evolve into a “knowledge-producing field,” which they define “as a domain of inquiry that includes academics from a range of disciplines as well as stakeholders not engaged in discipline-based knowledge production, such as professionals, administrators, and policymakers, all of whom focus on a common subject with the aim of advancing both theory and practice” (van Enk and Regehr 2018, p. 340). In this “knowledge-producing field,” academics and practitioners stand on equal footing and participate equally in the knowledge creation enterprise.

van Enk’s and Regehr’s article overlaps with ours as both examine aspects of interdisciplinary research. However, whereas they focus on the relationship between academics and practitioners, we centre our attention on the assumptions underlying the calls for an interdisciplinary turn in academia. Another point of divergence lies in the fact that van Enk’s and Regehr’s article is underpinned by a prescriptive orientation as they want members of the HPER field to modify their practices and behaviours in order to achieve a “well-functioning” (2018, p. 340) and “effective” research field (2018, p. 341). This goal might be achieved, van Enk and Regehr argue, if academic researchers and practitioners are “prepared to make concessions” (2018, p. 342) in their respective way of practicing research. Specifically, van Enk and Regehr advocate for a field in which the contribution of scientists will not take precedence over the contribution of non-scientists.

In contrast to van Enk and Regehr’s essay, we do not advocate for a particular direction in the evolution of the HPER field. Nor are we focused on the relationship between scientists and practitioners. Instead, we seek to develop a better understanding of the relationships between academic disciplines and interdisciplinarity with respect to the broader aim of knowledge production. Our aim is to inspire members of the HPER field (inclusive of scientists and practitioners) to reflect on the nature of their knowledge production and their connection with other research fields and disciplines.

## Challenging assumption 1: academic disciplines are silos

Let us begin by examining the assumption that disciplines are silos, which is a fundamental argument advanced by interdisciplinary promoters (see Table 1 for examples of this argument in the academic literature). If the assumption that academic disciplines are silos holds to be true, one would expect to see very little crossing of ideas, concepts, and methodologies from one discipline to another. Disciplines would be enclosed within rigid boundaries and academics would only be familiar with, and cite the work of their immediate fellow colleagues.

There are various ways to examine connections between disciplines. A common way in Science and Technology Studies (STS) is to look at citation patterns or perform network analysis (Jacobs 2014; Larivière and Gingras 2014; Leydesdorff et al. 2013; Wagner and Leydesdorff 2005). The data yielded by these methods allows one to map out and assess the knowledge flow across disciplines.

In our study, we draw on the technique used by Jacobs and Frickel (2009) and Jacobs (2014) in their recent work on interdisciplinarity. Similar to those studies, we used the Web of Science (WoS) database to examine citations patterns across diverse disciplines and research fields. Specifically, we explored whether concepts remain confined to the discipline or research field they originated from or whether concepts traveled across the academic landscape to other disciplines and knowledge domains. The concepts chosen for analysis are: “Actor Network Theory,” “Postmodernism,” and “Grounded Theory.” These three concepts were selected because they are known and used by HPER scholars with a social science background and they are sufficiently distinct that they will not be confused with other concepts.

The concept of Actor Network Theory was developed in the field of Science Studies by Bruno Latour, Michel Callon, and John Law in the 1980s. The concept of Postmodernism originated from the field of the Humanities and proliferated in the 1970s. The concept of Grounded Theory was developed by two medical sociologists, Barney Glaser and Anselm Strauss, in the 1960s. Using the WoS Core Collection database from Clarivate Analytics, three separate searches were done to trace the use of these concepts within and outside their originating fields.

First, “Actor Network Theory” was searched in title, abstract, or keywords of academic publications indexed in the WoS Core Collection using Topic Search (searches the title, abstract, keywords, and Keywords Plus fields). At the time of data collection, “Actor Network Theory” appeared in the title, abstract, or keywords, of 2702 academic publications in the WoS database. As Table 2 shows, the concept “Actor Network Theory” travelled well outside its field of origin of Science Studies and surfaced in disciplines and research areas as diverse as Anthropology, Computer Science, and Education. Table 2 includes research areas that represented 2% or more of the 2702 results. The WoS Categories presented are the subject category of its source publication, as categorized by WoS.

**Table 2:** The percentage and ranking of Web of Science Categories mentioning the concept “Actor Network Theory” (total articles = 2702). *Source*: Authors’ analysis of Web of Science Core Collection, Clarivate Analytics. Data compiled in June 2019

Rank	Web of Science Categories	Percent
1	Management	11.7
2	Sociology	11.4
3	Geography	9.4
4	Education Educational Research	8.4
5	Social Science Interdisciplinary	6.5
6	Computer Science Information Systems	5.9
7	Information Science Library Science	5.9
8	Business	5.8
9	Environmental Studies	5.6
10	Communication	4.8
11	History Philosophy of Science	3.5
12	Business Finance	3.1
13	Regional Urban Planning	2.8
14	Economics	2.8
15	Public Environment Occupational Health	2.8
16	Hospitality Leisure Sport Tourism	2.5
17	Cultural Studies	2.5
18	Computer Science Interdisciplinary Applications	2.3
19	Political Science	2.2
20	Social Issues	2.2
21	Anthropology	2.1
22	Social Science Biomedical	2.1

We repeated the analysis with the concept of Postmodernism, searching “Postmodern*” as a term in title, abstract, or keywords of academic publications indexed in the WoS Core Collection database (the asterisk was used to capture multiple ending variations such as Postmodernism, Postmodernity, etc.). “Postmodern*” appeared in the title, abstract, or keywords of 23,209 academic publications included in the WoS database. Our findings show a similar pattern as Latour et al’s “Actor Network Theory” concept: as Table 3 shows, “Postmodern*” has diffused well beyond the horizon of humanities scholarship. It has been used and discussed in a wide range of disciplines including Literature, Sociology, Education, and Management. Table 3 includes research areas that represented 2% or more of the 23,209 results.

**Table 3:** The percentage and ranking of Web of Science Categories mentioning the concept “Postmodern*” (total articles = 23,209). *Source*: Authors’ analysis of Web of Science Core Collection, Clarivate Analytics. Data compiled in June 2019

Rank	Web of Science Categories	Percent
1	Literature	12
2	Humanities	7.9
3	Religion	7.6
4	Philosophy	7.3
5	Sociology	6.7
6	Education Educational Research	6.6
7	Social Science Interdisciplinarity	4.4
8	Language Linguistic	3.8
9	Literary Theory Criticism	3.7
10	History	3.6
11	Political Science	3.4
12	Communication	2.7
13	Literature Romance	2.5
14	Geography	2.2
15	Cultural Studies	2.3
16	Management	2.1
17	Art	2.1

We repeated the same procedure with the concept “Grounded Theory.” The concept appears in the title, abstract, or keywords of 18,341 academic publications included in the WoS database. The diffusion pattern is similar to those of “Actor Network Theory” and “Postmodernism”: “Grounded Theory” travelled far outside the domain of Sociology where it was originally devised. Table 4 includes research areas that represented 2% or more of the 18,341 results.

**Table 4:** The percentage and ranking of Web of Science Categories mentioning the concept “Grounded Theory” (total articles = 18,341). *Source*: Authors’ analysis of Web of Science Core Collection, Clarivate Analytics. Data compiled in June 2019

Rank	Web of Science Categories	Percent
1	Nursing	13.5
2	Public Environmental Occupational Health	9.1
3	Education Educational Research	8.3
4	Social Sciences Interdisciplinary	7.9
5	Management	6.6
6	Health Care Sciences Services	6.0
7	Information Science Library Science	4.9
8	Social Sciences Biomedical	4.8
9	Rehabilitation	4.3
10	Business	4.3
11	Psychological Clinical	4.0
12	Psychiatry	3.8
13	Medicine General Internal	3.8
14	Education Educational Scientific Disciplines	3.3
15	Psychology Multidisciplinary	3.1
16	Computer Science Information Systems	3.1
17	Health Policy Services	2.9
18	Social Work	2.8
19	Family Studies	2.7
20	Psychology Applied	2.6
21	Sociology	2.4
22	Gerontology	2.1

As Tables 2, 3, and 4 show, each concept examined (Actor Network Theory, Postmodern*, Grounded Theory) was respectively used by academics in 22, 17, and 22 different research domains representing 2% or more of the citations. Altogether (after removing duplicates), the concepts were used in no less than 44 different domains. Based on these findings, one can make the claim that the current organisation of academic knowledge, predominantly structured around disciplines and research domains (Jacobs 2014), does not impede knowledge flow across the academic landscape. To the contrary, cross-pollination seems the norm across academic disciplines.

Larivière and Gingras (2014) also examined the claim of whether disciplines are silos. We reference this work, as their findings complement our own but do so over a much longer timeframe. Larivière and Gingras measured the degree of interdisciplinary of articles in three scientific domains from 1905 to 2005: medical science, social sciences, and the natural sciences and engineering. They created an interdisciplinary score for each article included in their dataset. This score is based on the number of references of each article to: (1) papers from other disciplines (e.g. Biology cited in Physics); (2) papers published from other specialities but in the same discipline (e.g. Optics cited in Nuclear Physics); and (3) papers published from the same specialties (e.g. Education cited in Education).

The highest percentage of interdisciplinary referencing is 50%, a score reached by the social sciences at the turn of the twentieth century and again more recently since 2000 (2014, p. 195, graph titled SS in Figure 10.4). Conversely, the lowest percentage of interdisciplinary referencing has been approximately 20%, an occurrence, still relatively high, found in the Natural Sciences and Engineering at the beginning of the twentieth century and again during the 1970s and 1980s (2014, p. 195, graph titled NSE in Figure 10.4). Medical Science positioned itself between these two poles, oscillating between 20 and 35% (2014, p. 195, graph titled MED in Figure 10.4). The longitudinal perspective offered by Larivière and Gingras’ data (from 1905 to 2005) shows that cross-disciplinary communication is far from being an ephemeral trend but is a rather well-established and longstanding practice among academic communities. While there have been variations across time in the intensity of cross-disciplinary knowledge exchange, it remains that scientists have always used the work from colleagues outside their own discipline to advance their research. Borrowing knowledge seems to be standard practice, not the exception. Further, this practice has been in place for more than 100 years, long before universities began reorganizing disciplinary structures.

Our data and those of Larivière and Gingras (2014) suggest too much knowledge exchange between disciplines to continue claiming that disciplines exist in silos and are inward looking. If disciplines are not siloed, by extension the argument for removing disciplinary barriers to facilitate knowledge flow across the academic landscape should also be re-examined. Scholars and scientists from all academic horizons spend a substantial part of their time reading, citing, using, and discussing the work of colleagues outside their discipline—a practice Rob Moore appropriately termed “routine interdisciplinarity” (2011). The amount of cross-disciplinary references is simply too substantial to be ignored. For this reason, interdisciplinary advocates must find a rationale other than the allegedly self-referential nature of disciplines to justify their desire to transform contemporary academia into a non (or less)-disciplinary institution. Empirical studies simply do not support their claim that the current organization of knowledge production creates insularity.

## Challenging assumption 2: interdisciplinary approaches are inherently better than discipline specific research approaches

Suppose that disciplines create artificial boundaries and offer a fragmented, and therefore impoverished, view of the natural and social world (assumption 1). Following this assumption (an assumption that is problematized by the data we have presented), the task facing interdisciplinary advocates is to reassemble this fragmented knowledge in a way that overcomes the purported disciplinary divisions. This is what interdisciplinary advocates endeavor to accomplish by implementing topic-centered research units across the university and supporting the creation of interdisciplinary research teams (Jacobs 2014; Moore 2011). In order to study complex problems, it is argued, disciplines need to give way to new and more flexible knowledge production arrangements. The knowledge gaps perceived to exist between disciplines need to be filled by interdisciplinary connections. These arrangements, by adding, and even blending, multiple perspectives are deemed to generate richer findings than would be possible within single disciplines. The assumption is that interdisciplinarity generates better research than single discipline research (Pizarro Milian and Missaghian 2018).

While we are obviously not against the idea of bringing together researchers from various fields to tackle complex problems, we question the taken-for-granted advantage of interdisciplinary over disciplinary research and the ensuing call for structural changes in academia. As Frickel et al. write: “A major problem that one confronts in assuming the superiority of interdisciplinary research is a basic lack of studies that use comparative designs to establish that measurable differences in fact exist and to demonstrate the value of interdisciplinarity relative to disciplinary research” (2017, p. 11). In other words, how can one affirm that interdisciplinary research generates more robust (more comprehensive, more sophisticated, etc.) knowledge than disciplinary research without any evidence that this is the case? As widespread as this belief may be, it remains an unsubstantiated assumption.

It would be a more balanced claim to argue that the value of research is determined by its fit to the nature of the problem being studied (Laberge et al. 2009). Neither disciplinary nor interdisciplinary approaches can be said to be intrinsically better than the other, as their respective merit is dependent on the nature of the phenomenon under investigation (e.g., understanding environmental issues will likely necessitate the input of multiple disciplines from the natural and social sciences, while measuring the efficacy of a medical treatment may likely be comprehensively achieved through a clinical epidemiological study).

Both forms of research (disciplinary and interdisciplinary) have in fact always coexisted and been used complementarily, and disciplines have coalesced as needed to form new research fields. Disciplines are not petrified or insular as interdisciplinarians commonly argue (see Table 1 for examples of these arguments) but are in constant flux and have both fuzzy and porous boundaries (Frickel et al. 2017; Jacobs 2014; Moore 2011). For example, Cognitive Science developed as an interdiscipline during the 1950s by combining Computer Science, Linguistics, Neuroscience, Philosophy, and Psychology (Graff 2015). Social Epidemiology emerged during the second half of the twentieth century at the intersection of Epidemiology and Sociology (Berkman and Kawachi 2000). The sociologist Pierre Bourdieu developed his theory by creatively drawing on the disciplines of Sociology, History, Anthropology, and Linguistics (the latter for his work on language) (Medvezt and Sallaz 2018). Disciplinary and interdisciplinary research are not opposed to one another and stating that one is better than the other is at best an unfounded value judgment until proven otherwise.

It follows from these observations that top-down policies and organizational arrangements promoting collaborations across disciplines—instead of letting academics freely collaborate based on their quest for understanding and finding answers to pressing problems—could be unproductive. If academics already do, at least to some extent, what interdisciplinary advocates argue they should do, what is the rationale for, and the interest behind, the call for organizational changes to facilitate cross-disciplinary collaboration? This question is beyond the scope of this article, but needs continued attention as it raises considerations about academic freedom and who holds the authority to decide what research problems should be studied and how (Albert et al. 2007; Albert et al. 2017a; Jacobs 2014; Laberge et al. 2009; Moore 2011).

## Challenging assumption 3: interdisciplinarity overcomes fragmentation

Another assumption to be considered by interdisciplinary advocates is whether interdisciplinarity overcomes disciplinary fragmentation or simply re-divides knowledge fields along new demarcation lines. The ambition to create a boundary-free research space by erasing the disciplines may result in displacing boundaries rather than removing them (Albert et al. 2017b; Jacobs 2014; Moore 2011). Indeed, an academic environment structured around topic-centered research areas (e.g., global health, simulation, interprofessional education) would be as fragmented, if not more, than a disciplinary-structured environment. While there is a relatively finite number of disciplines (Sociology, Psychology, Chemistry, etc.), the number of topics is innumerable and change constantly as new issues appear, further fragmenting and re-fragmenting the research landscape (Abbott 2001; Jacobs 2014).

Disciplines, in contrast, are less prone to fall into the fragmentation spiral. They usually grow out from a set of foundational assumptions which ensure continuity over time. These foundational assumptions could be described as ways of seeing and conceptualising the world, and ways of asking questions about phenomena. For example, contemporary sociology, despite its numerous theoretical ramifications and internal sub-specialties (e.g., Symbolic Interactionism, Ethnomethodology, Structuro-functionalism, Sociology of Education, Medical Sociology, Sociology of the Professions) still draw on the foundational assumptions developed by Emile Durkheim ([1894] 2014) and Max Weber ([1922] 2019) in the 19th and early 20th centuries. These assumptions are that individuals’ behaviour can be *explained* by looking at social factors (Durkheim’s assumption ([1894] 2014)) and *understood* by probing the meaning social actors attribute to their actions and social environment (Weber’s assumption ([1922] 2019)). Sociology has undergone continuous transformations but has not dismissed Durkheim’s and Weber’s foundational assumptions. Contemporary sociologists may work on topics and use theories unknown to their predecessors, but they still build their work on the assumption that behavioral patterns are shaped by the culture of the social group that one is embedded in.

In contrast, it is uncertain that topic-centered research areas, such as patient safety or interprofessional education, will be able to maintain their existence if health care organisations and funders cease to see the need to support them and redirect resources elsewhere. In the absence of any foundation other than being useful to address current social issues, they would likely disappear while other topics would emerge, furthering the cycle of fragmenting and re-fragmenting of the HPER landscape. Considering this, it is unclear how the multiplication of interdisciplinary topic-centered research centres can be a solution to the purported problem of knowledge fragmentation allegedly created by the disciplines.

Interdisciplinary research set up as topic-centered research communities may create another line of demarcation. Over time, researchers may predominantly engage in conversation with peers who share the same topic interest. Successful interdisciplinary research communities may then come to emulate established disciplines by developing their own journals, conferences, professional associations, and tendency to hire from within. These communities would reproduce discipline-like boundaries as Jacobs noted (2014), resulting in the same kind of alleged silos interdisciplinary advocates sought to breakdown. Following this reasoning, the interdisciplinary ideal of breaking down (imaginary) silos by restructuring the academy around topic-centered units may contain the seed of its own failure.

## Implications for the field of health professions education research

Having now explored three underlying assumptions animating the argument for interdisciplinarity in research more broadly, we turn to the field of HPER more specifically. In this section, we argue that the field of HPER has largely adopted the premises for why interdisciplinary research is necessary but has not yet debated the potential effects of organizing around these premises.

The adoption of the premises for the necessity of interdisciplinary research has manifested in various features of HPER. For example, training programs and research activities are commonly defined in terms of methodologies (qualitative versus quantitative methods) as opposed to disciplines, therefore skirting disciplinary knowledge. In the “Aims and Scopes” posted on their websites, HPER journals typically describe their publication interest in terms of topics (e.g. curriculum development, clinical reasoning, interprofessional education) and level of medical training (undergraduate education, postgraduate training, continuing professional development), leaving out any notion regarding the disciplines (e.g. Academic Medicine 2019; Advances in Health Sciences Education n.d.; Medical Education 2019). Several leading HPER research centres and departments profile themselves as being interdisciplinary (e.g., the School of Health Profession Education, Maastricht University (n.d.); the Centre for Education Research & Innovation, Western University (2019); the Centre for Medical Education, McGill University (2019)). A pilot study conducted by the principal author (MA) found that HPER scholars themselves downplay disciplinary knowledge as they tend to rarely cite work from disciplinary journals. Only 2% of 726 references cited in 20 research articles published in 2017 from two high profile HPER journals came from disciplinary journals (Albert 2018). Researchers in the field typically introduce themselves as either a qualitative or quantitative researcher, even those with disciplinary training. This binary is part of what one could call the doxa of the field (Bourdieu 1987), i.e., the internalised and therefore taken-for-granted cognitive categories through which research practices are conceived. Further, a lack of scholarly work in HPER on the topic of interdisciplinarity (with the recent exception of van Enk and Regehr 2018) suggests that the field seems to have tacitly endorsed the assumptions underpinning interdisciplinary research without careful consideration of the benefits and costs of such endorsement. While disciplinary-based knowledge is scarce in HPER, this scarcity seems to be the result of an unspoken convention rather than of a deliberate decision. Based on these observations, it appears that disciplinary knowledge and disciplinary schemes for categorizing research practices do not easily make their way into the HPER field.

The examination of the assumptions associated with interdisciplinarity introduced earlier has further implications for how one thinks about the field of HPER. First, we suggest that retaining the pro-interdisciplinary claim that one should be wary of disciplines because they hinder intellectual exchange would be taking a position inconsistent with the evidence. The examination of how concepts travel across the academic landscape demonstrates that the silo thesis does not hold up against bibliometric data. It is also unfounded to posit that interdisciplinarity necessarily enhances knowledge making by creating a new, boundary-free academic terrain.

Thus, what would be the rationale for the HPER community to continue defining knowledge production in a way that echoes (yet, more by habit than through careful consideration and choice) the interdisciplinary refrain? What is the rationale for using the categories of methodological preference (qualitative versus quantitative research), topics (curriculum development, clinical reasoning, interprofessional education), and training levels (undergraduate education, postgraduate training, continuing professional development), without making any reference to, and drawing upon, disciplinary knowledge? If HPER cannot invoke limitations created by the disciplines to justify current practices, what is the value-added for scholars to situate their research practices outside the disciplines? We believe it would benefit HPER scholars to collectively engage in reflection about HPER research practices and bring to light the tenets which currently orient knowledge making in the field.

Furthermore, we believe it is legitimate to ask whether HPER risks becoming insular if it cuts ties with the disciplines. If so, HPER scholars might be confined to the rediscovery of what is already known outside the field. Likewise, how can HPER advance theoretical knowledge without drawing on theories developed in the disciplines and in the established research areas such as Higher Education Studies and Science Studies? Relatedly, why would researchers from the broader academic fields and disciplines pay attention to HPER if HPER researchers neglect to engage in a conversation with disciplines and show the value of HPER contributions to the broader pool of academic knowledge? These questions are not solely rhetorical; they matter when HPER researchers find themselves in competition with other disciplines for access to resources, for example at national funding agencies—such as the Tri Councils in Canada or the National Science Foundation in the United States—governments, and health care organisations. Ultimately, these questions concern HPER’s place and scientific credibility within the broader academic market. As a growing, but still small community, HPE researchers should not be blind to the potential insularity that could come from continued positioning outside the disciplines. Such positioning may inadvertently undermine—rather than strengthen—the HPER field.

With this article, our hope is to inspire members of the HPER community to critically examine the ubiquitous discourse promoting interdisciplinarity and engage in reflection about the future of the field informed by evidence rather than by unsubstantiated assumptions. Should research centres and graduate programs in HPER encourage the development of interdisciplinary or disciplinary-trained researchers? Should training predominantly focus on methods and methodologies or draw more on disciplinary-based knowledge? What is the best route toward increasing the field’s profile within academia and attracting the best students and researchers to engage in HPER? How can HPER meaningfully participate in debates occurring across the academy about disciplines and interdisciplinarity and play a role in shaping higher education and research policies? These are the questions that merit attention at the current juncture. The future of the HPER field relies on decisions made in the present time. It behooves the field to carefully consider the assumptions built into current arguments regarding knowledge production, as these assumptions have effects on HPER training, and the nature of the knowledge generated in the field.

## References

[CR1] Abbott A (2001). Chaos of disciplines.

[CR2] Academic Medicine. (2019). *About the journal: Academic Medicine*. Retrieved January 14, 2019 from https://journals.lww.com/academicmedicine/Pages/journaltimeline.aspx.

[CR3] Advances in Health Sciences Education. (n.d.) *Advances in Health Sciences Education*—*Aims and Scope.* Retrieved January 14, 2019 from https://www.springer.com/education+%26+language/journal/10459.

[CR4] Albert, M. (2018). *Intersections, introspections and divergences: sustaining the growth of medical education research and training*. AMEE Symposium presented at an International Association for Medical Education (AMEE) Conference, Basel, Switzerland.

[CR5] Albert M, Hodges B, Regehr G (2007). Research in medical education: balancing service and science. Advances in Health Sciences Education.

[CR6] Albert M, Paradis E, Kuper A (2017). Interdisciplinary promises versus practices in medicine: The decoupled experiences of social sciences and humanities scholars. Social Sciences & Medicine.

[CR7] Albert M, Paradis E, Kuper A, Frickel S, Albert M, Prainsack B (2017). Interdisciplinary fantasy: Social scientists and humanities scholars working in faculties of medicine. Investigating interdisciplinary collaboration: Theory and practice across disciplines.

[CR8] Armstrong PW (2006). Advancing interdisciplinary health research: a synergism not to be denied. Canadian Medical Association Journal (CMAJ).

[CR9] Berkman LF, Kawachi I, Berkman L, Kawachi I, Glymour M (2000). A historical framework for social epidemiology. Social epidemiology.

[CR10] Bourdieu P (1987). Choses dites.

[CR11] Brint S (2005). Creating the future: ‘New directions’ in American research universities. Minerva.

[CR12] Durkheim, E. ([1894] 2014). *The rules of sociological method: and selected texts on sociology and its method*. Simon and Schuster.

[CR13] Frickel S, Albert M, Prainsack B, Frickel S, Albert M, Prainsack B (2017). Introduction: Investigating interdisciplinarity. Investigating interdisciplinary collaboration: Theory and practice across disciplines.

[CR14] Giacomini M (2004). Interdiscplinarity in health in health services research: Dreams and nightmare, maladies and remidies. Journal of Health Services Research & Policies.

[CR15] Graff H (2015). Undisciplining knowledge: Interdisciplinarity in the twentieth century.

[CR16] Jacob, W. J. (2015). Interdisciplinary trends in higher education. *Palgraves Communication 1*.

[CR17] Jacobs JA (2014). In defense of disciplines interdisciplinarity and specialization in the research university.

[CR18] Jacobs JA, Frickel S (2009). Interdisciplinarity: A critical assessment. Annual Review of Sociology.

[CR19] Laberge S, Albert M, Hodges BD (2009). Perspectives of clinician and biomedical scientists on interdisciplinary health research. Canadian Medical Association Journal (CMAJ).

[CR20] Larivière V, Gingras Y, Cronin B, Sugimoto C (2014). Measuring interdisciplinarity. Beyond bibliometrics: Harnessing multidimensional indicators of scholarly impact.

[CR21] Leydesdorff L, Rafolls I, Chen C (2013). Interactive overlays of journals and the measurement of interdiscplinarity on the basis of aggregated journal–journal citations. Journal of the American Society of Information Science and Technology.

[CR22] van Enk A, Regehr G (2018). HPE as a field: Implications for the production of compelling knowledge. Teaching and Learning in Medicine.

[CR23] Maastrich University. (n.d.). School of Health Professions Education. Retrieved from https://she.mumc.maastrichtuniversity.nl/contact-she

[CR24] McGill University. (2019). Centre for Medical Education. Retrieved from https://www.mcgill.ca/centreformeded/

[CR25] Medical Education. (2019). Overview—Medical education. Retrieved January 14, 2019. https://onlinelibrary.wiley.com/page/journal/13652923/homepage/productinformation.html.

[CR26] Medvezt T, Sallaz JJ (2018). The Oxford handbook on Pierre Bourdieu.

[CR27] Moore R, Christie F, Maton K (2011). Making the break: Disciplines and interdisciplinarity. Disciplinarity: Functional linguistic and sociological perspectives.

[CR28] National Academy of Sciences (2005). Facilitating interdisciplinary research.

[CR29] Organization for Economic Co-operation and Development (OECD) (1972). Interdisciplinarity: Problem of teaching and research in universities.

[CR30] Pizarro Milian R, Missaghian R (2018). Interdisciplinarity for sale: Logics of knowledge, labour markets and consumerism. Higher Education Quarterly.

[CR31] Sá CM (2008). ‘Interdisciplinary strategies’ in US research universities. Higher Education.

[CR32] Townsend T, Pisapia J, Razzaq J (2015). Fostering interdisciplinary research in universities: A case study of leadership, alignment and support. Studies in Higher Education.

[CR33] Wagner CS, Leydesdorff L (2005). Network structure, self-organization and the growth of international collaboration in science. Research Policy.

[CR34] Weber, M. ([1922] 2019). *Economy and society*. Cambridge, Mass. Harvard University Press.

[CR35] Western University. (2019). Centre for Education Research and Innovation. Retrieved from https://www.schulich.uwo.ca/ceri/

